# Synthesis and NMR-Study of the 2,3,4,5-Tetraethylsilole Dianion [SiC_4_Et_4_]^2−^•2[Li]^+^

**DOI:** 10.3390/molecules16098033

**Published:** 2011-09-16

**Authors:** Jang-Hwan Hong

**Affiliations:** Department of Nanopolymer Material Engineering, Pai Chai University, 155-40 Baejae-ro (Doma-Dong), Seo-Gu, Daejon 302-735, Korea; Email: jhong@pcu.ac.kr; Tel.: +82-42-520-5755; Fax: +82-42-520-5798

**Keywords:** silole, germole, dianion, group 14 metallole, aromaticity

## Abstract

The previously unknown silole dianion [SiC_4_Et_4_]^2−^•2[Li]^+^ (**3**) was prepared by the sonication of 1,1-dichloro-2,3,4,5-tetraethyl-1-silacyclopentadiene [Cl_2_SiC_4_Et_4_, **2**] with more than four equivalent of lithium in THF. ^1^H-, ^13^C-, and ^29^Si-NMR data of **3** are compared with those of the reported silole dianion [SiC_4_Ph_4_]^2−^. Trapping of **3** with trimethylchlorosilane gave 1,1-bis(trimethylsilyl)-2,3,4,5-tetraethyl-1-silacyclopentadiene [(Me_3_Si)_2_SiC_4_Et_4_, **4**] in high yield. The silole of **2** was synthesized in high yield in three steps by a modified procedure using Cp_2_ZrCl_2_ *via* Cp_2_ZrC_4_Et_4_ and 1,4-dibromo-1,2,3,4-tetraethyl-1,3-butadiene.

## 1. Introduction

Since the first silole dianion, 2,3,4,5-tetraphenyl-1-silacyclopentadienide dianion, was prepared in 1990 by Joo and Hong [1], the aromaticity of the silole dianion [2] and germole dianion [3] was suggested by NMR study and it was confirmed by X-ray crystallography of the structures [4,5,6,7,8] and by theoretical study [9,10]. The chemistry of group 14 metallole dianions has been developed enormously [11,12], and recently the stannole dianion [SnC_4_Ph_4_]^2−^ was also reported [13,14,15,16].

**Figure 1:**
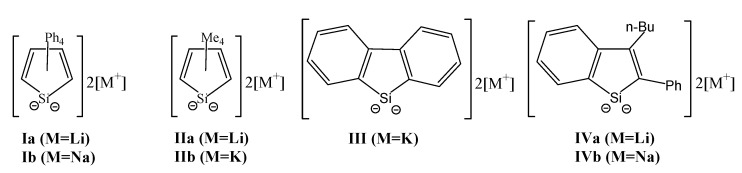
Silole dianions, silafluorenyl dianions, and silaindenyl dianions.

In contrast, only two silole dianions have been reported so far; [SiC_4_Ph_4_]^2−^ (**I**) [1,2,4], [SiC_4_Me_4_]^2−^ (**II**) [5] with the silafluorenyl dianion [SiC_4_(CH_2_)_8_]^2−^2[M]^+^ (**III**) [17,18] and the silaindenyl dianion [(CH_2_)_4_C_2_SiC_2_PhBu]^2−^•2[M]^+^ (**IV**) [19] since the available 1,1-dihalosiloles are limited (Figure 1).

Only two silole dianions are reported since the synthetic methods for 1,1-dihalosiloles are limited to 1,1-dichloro-2,3,4,5-tetraphenyl-1-silacyclopentadiene, 1,1-dibromo-2,3,4,5-tetramethyl-1-silacyclopentadiene, and 1,1-dichloro-2,3,4,5-tetrametyl-1-silacyclopentadiene. The former is readily prepared from SiCl_4_ and 1,4-dilithio-2,3,4,5-tetraphenyl-1,3-butadiene, which is easily produced from diphenylacetylene and lithium, however, it is unable to exchange the phenyl groups with other groups [1]. 1,1-Dibromo-2,3,4,5-tetramethyl-1-silacyclopentadiene is synthesized from Cp_2_ZrC_4_Me_4_ and SiBr_4_ in very low yield [20]. 1,1-Dichloro-2,3,4,5-tetrametyl-1-silacyclopentadiene is synthesized from 1,4-diiodo-1,2,3,4-tetramethyl-1,3-butadiene via Cp_2_ZrC_4_Me_4_ [21]. Here we report the synthesis of 1,1-dichloro-2,3,4,5-tetraethyl-1-silacyclopentadiene [Cl_2_SiC_4_Et_4_] and an NMR study of the silole dianion [SiC_4_Et_4_]^2−^•2[Li]^+^.

## 2. Results and Discussion

**Scheme 1:**
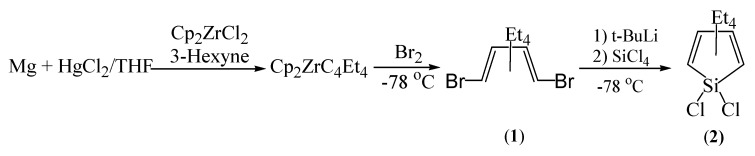
Synthesis of 1,1-dichloro-2,3,4,5-tetraethyl-1-silacyclopentadiene (**2**).

We have prepared 1,4-dibromo-1,2,3,4-tetraethyl-1,3-butadiene (**1**) by a modified procedure using Cp_2_ZrCl_2_ [22] and bromine (Scheme 1).

**Scheme 2:**
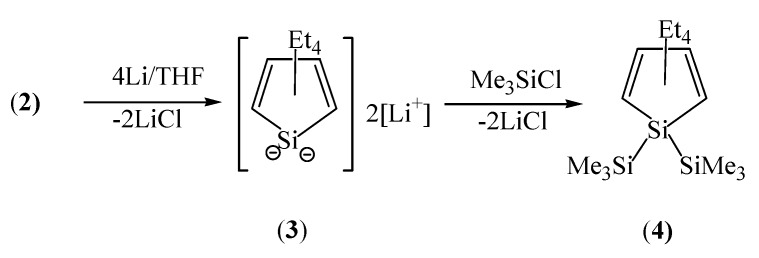
Synthesis of 1,1-bis(trimethylsilyl)-2,3,4,5-tetraethyl-1-silacyclopentadiene (**4**).

Addition of SiCl_4_ to 1,4-dilithio-1,2,3,4-tetraethyl-1,3-butadiene, which is obtained by the metallation of **1** by *t*-BuLi, gives a 75% yield of pure 1,1-dichloro-2,3,4,5-tetraethyl-1-silacyclopentadiene [Cl_2_SiC_4_Et_4_, **2**]. It has been previously reported that 1,4-diioodo-1,2,3,4-tetraethyl-1,3-butadiene and **2** have been isolated only as impure materials [8]. Sonication of **2** with more than four equivalents of lithium in THF produces a dark red solution. Trapping of **3** with trimethylchlorosilane provides [(Me_3_Si)_2_SiC_4_Et_4_, **4**] in 95% yield (Scheme 2).

The NMR study of the red solution in THF-*d*_8_ shows the only one species, which is assigned to the structure **3**. The ^13^C-NMR spectrum of **3** presents six peaks, consistent with *C*_2_ symmetry, and the ^29^Si spectrum of **3** shows only one resonance. Upon lithiation of **2** to **3**, the ^29^Si resonance is shifted downfield (Δδ = 16.66 ppm, 8.30 ppm for **2** and 24.96 ppm for **3**, Table 1) and the ^13^C resonances of C_α_ and C_β_ are shifted upfield compared with **2** {Δδ(C_α_) = −4.84 and Δδ(C_β_) = −3.55)} [23] (Table 2).

**Table 1:** ^29^Si Chemical shifts.

2 ^a^	3 ^b^	Ia ^b^	Ib ^b^	IIa ^c^	III ^b^	IVa ^b^	IVb ^b^
8.30	24.96	68.54	92.79	29.77	29.00	29.19	30.44

^a^ In CDCl_3_, reference; external TMS as standard; ^b^ In THF-*d*_8_, reference = 25.30 ppm; ^c^ In THF-*d*_8_, reference; external TMS as standard.

**Table 2:** ^13^C-NMR chemical shifts.

	2 ^a^		3 ^b^		4 ^a^			3-2	
C_α_	155.48		150.64		155.03		Δδ(C_α_)	−4.84	
C_β_	130.59		127.04		139.36		Δδ(C_β_)	−3.55	
T	572.17		555.36		588.78		ΔT	−16.78	
	α Et	β Et	α Et	β Et	α Et	β Et		α Et	β Et
C1	20.82	20.56	22.24	26.28	21.38	22.83	ΔC1	1.42	5.72
C2	14.37	14.06	19.59	21.89	15.24	16.88	ΔC2	5.22	7.83

^a^ In CDCl_3_, reference; external TMS as standard; ^b^ In THF-*d*_8_, reference = 25.30 ppm.

These chemical shifts of ^29^Si and ^13^C resonances are consistent with delocalization of the negative charge into the silole ring, which is supported by the calculated negative NICS value of dilithiumsilole dianion [9,10]. In addition the signals of the ethyl groups in the ^1^H- and ^13^C-NMR spectra of **3** shift downfield due to the anisotropic effect of the ring current from the delocalization {Δδ(^13^C of CH_2_CH_3_) = 1.42–7.83 ppm and Δδ(^1^H of CH_2_) = 0.16–0.19 ppm)} (Table 3).

**Table 3:** ^1^H-NMR chemical shifts.

	2 ^a^		3 ^b^		4 ^a^			3-2	
	α Et	β Et	α Et	β Et	α Et	β Et		α Et	β Et
CH_2_	2.31	2.34	2.50	2.50	2.34	2.36	ΔC1	0.19	0.16
CH_3_	1.03	1.18	1.13	1.13	0.99	1.05	ΔC2	0.10	−0.05

^a^ In CDCl_3_, reference; external TMS as standard; ^b^ In THF-*d*_8_, reference = 1.73 ppm.

Surprisingly, the chemical shift of ^29^Si resonance in **3** is similar to those of **IIa**, **III**, and **IV**, even though **III** and **IV** have conjugated benzene rings on the silole rings (Table 1). In addition the chemical shifts of C_α_ and C_β_ (150.64 and 127.04 ppm) in **3** are very close to those of the reported tetraphenyl substituted silole dianions {Δδ(C_α_) = 0.58 and Δδ(C_β_) = 2.71 ppm for [SiC_4_Ph_4_]^2−^•2[Li]^+^(**Ia**) and Δδ(C_α_) = 3.10 ppm and Δδ(C_β_) = 3.88 ppm for [SiC_4_Ph_4_]^2−^•2[Na]^+^**(Ib)**} even if **3** has four ethyl groups on the ring. However, the chemical shifts of C_α_ and C_β_ (150.64 and 127.04 ppm) in **3** are quite different from those of C_α_ and C_β_ (138.97 ppm and 119.97 ppm) in **IIa** [21]. These data unambiguously indicate that four phenyl groups on the ring have no conjugation with the butadiene ring as shown by X-ray crystallography [4] and instead, the π-polarization of the phenyl groups on the ring is observed in **I** due to the increased electron density on the ring [2] (Table 4).

**Table 4:** ^13^C-NMR chemical shifts.

	[Cl_2_SiC_4_Ph_4_] ^a^[1]		Ia ^b^[2]		Ib ^b^[1]	
C_α_	154.74		151.22		153.74	
C_β_	132.28		129.71		130.92	
T	574.04		561.86		569.32	
	α Ph	β Ph	α Ph	β Ph	α Ph	β Ph
C_i_	136.67	135.37	151.67	145.83	151.29	146.71
C_o_	139.48	129.27	129.97	133.43	129.48	133.16
C_m_	127.84	128.24	126.38	126.38	126.55	126.72
C_p_	127.37	127.10	119.48	121.83	118.25	121.42
C_i_-C_p_	7.00	8.27	32.19	24.00	33.04	25.29
Sum (C_i_-C_p_)	17.57		56.19		58.33	

^a^ In CDCl_3_, reference; external TMS as standard; ^b^ In THF-*d*_8_, reference = 25.30 ppm.

The ^29^Si chemical shift for **3** at 24.96 ppm shifts downfield comparing to 8.30 ppm for **2**, however the chemical shift is more downfield than those of the tetraphenyl substituted silole dianion [SiC_4_Ph_4_]^2−^•2[M]^+^ (68.54 ppm for M = Li, **Ia** and 92.79 ppm for M = Na, **Ib**).

In ^31^P-NMR of the phosphoryl anion, which is isoelectronic with the silole dianion, the same downfield chemical shifts are observed [24]. The large downfield shifts of the phosphoryl anions have been ascribed to the conjugation effect of p-π orbital electrons and to the presence of the in-plane lone pair weakly coupled to the ring [25,26]. This paramagnetic shift depends on the narrow energy gap between HOMO and LUMO. The smaller gap is between HOMO and LUMO, the more paramagnetic shielding is assigned to the NMR chemical shifts [27]. If the in-plane nonbonding orbital is the HOMO, the energy level of the HOMO is less affected by the substituents of the butadiene moiety relatively. However LUMO greatly depends on the substituents of the butadiene moiety since the LUMO is one of the anti-bonding MOs of the 5-membered ring. Therefore the LUMO of [SiC_4_Ph_4_]^2−^•2[Li]^+^ should be stabilized relatively compared to that of [SiC_4_Ph_4_]^2−^•2[Li]^+^ by the effect of the substituents on the butadiene moiety or *vice versa*. This rationale is reinforced by the comparison of the electronegativities between the phenyl and the ethyl groups (the phenyl group has higher electronegativity than the ethyl group, 2.717 and 2.481, respectively [28]. The difference between ^29^Si chemical shifts of [SiC_4_Ph_4_]^2−^ and [SiC_4_Et_4_]^2−^ might be due to the paramagnetic shielding effect of the substituents on the silole ring.

## 3. Experimental

### General Procedures

All reactions were performed under an inert nitrogen atmosphere using standard Schlenk techniques. Air sensitive reagents were transferred in a nitrogen-filled glovebox. THF and ether were distilled from sodium benzophenone ketyl under nitrogen. Hexane and pentane were stirred over concentrated H_2_SO_4_ and distilled from CaH_2_. NMR spectra were recorded on JEOL GSX270 and GSX400 spectrometers. GC-MS and solid sample MS data were obtained on a Hewlett-Packard 5988A GC-MS system equipped with a methyl silicon capillary column. Elemental analyses were done by Desert Analytics (Tucson, AZ, USA).

*1,4-Dibromo-1,2,3,4-tetraethyl-1,3-butadiene* (1). The synthetic procedures for the preparation of Cp_2_ZrC_4_Et_4_ are modified from the known procedures [20]. A mixture of Mg (7.78 g, 320 mmol) and HgCl_2_ (8.69 g, 32 mmol) in THF (100 mL) was stirred for 1 h, to this was added a solution of Cp_2_ZrCl_2_ (23.4 g, 80 mmol) and 3-hexyne (18.14 mL, 160 mmol) in THF (250 mL) with stirring at room temperature. Stirring overnight gave a dark red solution. The solvent was removed under reduced pressure, and the red-orange residue was extracted with hexane. Removal of the hexane yielded a red-orange solid of pure Cp_2_ZrC_4_Et_4_ (27.6 g, yield 90%). Bromine (7.40 mL, 143 mmol) was slowly added to Cp_2_ZrC_4_Et_4_ (27.6 g, 71.5 mmol) in ether (300 mL) at −78 °C with stirring. After it was stirred for 1 h, the mixture was warmed up to room temperature. The reaction mixture was filtered and the filtrate was treated with the saturated aqueous Na_2_S_2_O_3_ solution. The organic layer was separated, dried with Na_2_SO_4_, filtered and distilled do give 1,4-dibromo-1,2,3,4-tetraethyl-1,3-butadiene. Yield, 16.2 g (70%, purity; 99% by GC), bp 110–125 °C/0.1 mmHg; ^1^H-NMR (CDCl_3_, ref; ext. TMS = 0.00 ppm), 1.08 (t, Me, 6H, *J *= 7.33 Hz), 1.16 (t, 6H, Me, *J* = 7.33 Hz), 2.05–2.22 (m, 2H, CH_2_), 2.30–2.42 (m, 2H, CH_2_), 2.42–2.65 (m, 4H, CH_2_); ^13^C-NMR (CDCI_3_, ref; solvent = 77.00 ppm), 140.13 (C1), 126.47 (C2), 30.70 (CH_2_ of C1), 25.60 (CH_2_ of C2), 13.18 (Me of C1), 12.89 (Me of C2); MS(M^+^, relative abundance), 266 (M^+^+4, 5), 265(M^+^+3, 4), 264 (M^+^+2, 18), 263 (M^+^+1, 6), 262 (M^+^, 27), 235 (M^+^-27, 16), 233 (M^+^−29, 21), 164 (C_4_Et_4_^+^, 100), 149 (C_4_Et_4_^+^−15, 55), 135 (95), 107(42).

*[Cl_2_SiC_4_Et_4_]* (2). To 1,4-dibromo-1,2,3,4-tetraethyl-1,3-butadiene (11.8 g, 36.41 mmol) in ether (200 mL) was added *t*-BuLi in hexane (64 mL, 1.7 M, 109.2 mmol) at −78 °C. After it was stirred for 2 h, to it was added SiCl_4_ (11.51 mL, 109.2 mmol) with stirring at −78 °C. The mixture was warmed up to room temperature, and stirred overnight to produce a clear yellow solution. After the solvent was removed under reduced pressure, the remaining solid was extracted with pentane. Distillation of pentane under reduced pressure gave a colorless liquid. Yield, 7.0 g (74%, purity; 99% by GC), bp 140–160 °C under aspirator pressure; MS (M^+^, relative abundance), 327 (M^+^+5, 1), 326 (M^+^+4, 9), 325 ((M^+^+3, 2), 324 (M^+^+2, 20), 323 (M^+^+1, 1), 322 (M^+^, 10), 245 (M^+^−81, 68), 243 (M^+^−79, 68), 164 (C_4_Et_4_^+^, 45), 163 (C_4_Et_4_^+^−1, 45), 149 (C_4_Et_4_^+^−15, 38), 135 (61), 107 (100). Anal Calcd. for C_12_H_20_SiCl_2_: C, 54.74; H, 7.66, Found: C, 54.50; H, 7.69.

*[(Me_3_Si)_2_SiC_4_Et_4_]* (4). Sonication of 2 (1.26 g, 4.79 mmol) and lithium (0.15 g, 21.43 mmol) for 12 h gave a dark red solution. After filtration, it was added to an excess of trimethylchlorosilane (2.0 mL, 15.80 mmol). Stirring for 2 h at room temperature produced a pale brown solution immediately. All volatiles were removed under reduced pressure, and the residue was extracted with hexane. Evaporation of the hexane gave a colorless oil. It had been previously reported [8] that the product 4 was obtained in 45% yield. Yield; 95% by ^1^H-NMR integration; ^1^H-NMR (CDCl_3_, ref; ext. TMS = 0.00 ppm), 0.14 (s, SiMe_3_, 18H), 0.99 (t, 6H, Me, *J* = 7.33 Hz), 1.05 (t, 6H, *J* = 7.33 Hz), 2.34 (q, 4H, CH_2_, *J* = 7.33 Hz), 2.36 (q, 4H, CH_2_, *J* = 7.33 Hz);^13^C-NMR (CDCI_3_, ref; solvent = 77.00 ppm), −0.08 (SiMe); ^29^Si-NMR (THF-*d*_8_, ref; ext. TMS = 0.00 ppm), −14.99 (ring Si), −38.04 (SiMe_3_); MS (M^+^, relative abundance), 340 (M^+^+2, 2), 339 (M^+^+1, 5), 338 (M^+^, 13), 309 (M^+^−29, 2), 267 (M^+^+2−73, 4), 266 (M^+^+1−73, 11), 265 (M^+^−73, 38), 235 (3), 73 (100), 59 (36); Anal Calcd for C_18_H_38_Si_3_: C, 63.82, H, 11.31, Found: C, 63.73; H, 11.54.

*[SiC_4_Et_4_]^2−^•2[Li]^+^*; Sonication of **2** (0.12 g, 0456 mmmol) and lithium (0.020 g, 2.857 mmmol) in 1.5 mL of THF-*d*_8_ for 6 h gave a dark red solution.

*[SiC_4_Ph_4_]^2−^•2[M]^+^* (M = Li, Na); It was prepared according to the known procedure [1,2].

## 3. Conclusions

1,1-Dichloro-2,3,4,5-tetraethyl-1-silacyclopentadiene (**2**) is prepared from SiCl_4_ and 1,4-dilithio-1,2,3,4-tetraethyl-1,3-butadiene, the precursor of which, 1,4-dibromo-1,2,3,4-tetraethyl-1,3-butadiene (**1**), is synthesized from 3-hexyne and Cp_2_ZrCl_2_. Sonication of the silole **2** with an excess of lithium in THF produces the silole dianion [SiC_4_Et_4_]^2−^•2[Li]^+^(**3**), the treatment of which with trimethylchlorosilane gives 1,1-bis(trimethylsilyl)-2,3,4,5-tetraethyl-1-silacyclopentadiene (**4**). The NMR study of **3** shows that the ^29^Si resonance in **3** shifts downfield and the ^13^C resonances of C_α_ and C_β_ in **3** shift upfield compared with **2**. In particular both chemical shifts of C_α_ and C_β_ in **3** are very close to those of the reported tetraphenyl-substituted silole dianions, [SiC_4_Ph_4_]^2−^•2[Li]^+^ and [SiC_4_Ph_4_]^2−^•2[Na]^+^.
